# The Glutathione Peroxidase Gene Family in *Chenopodium quinoa*: Genome-Wide Identification, Classification, Gene Expression and Functional Analysis

**DOI:** 10.3390/antiox14080940

**Published:** 2025-07-30

**Authors:** Jing Yang, Anna Xu, Kexin An, Lilong Wang, Taiping Luo, Xinyue Yu, Haibo Yin, Shanli Guo, Xia Zhang

**Affiliations:** Yantai Key Laboratory of Characteristic Agricultural Bioresource Conservation & Germplasm Innovative Utilization, School of Life Sciences, Yantai University, Yantai 264005, China; yangjing@s.ytu.edu.cn (J.Y.); yhb@ytu.edu.cn (H.Y.)

**Keywords:** *Chenopodium quinoa*, glutathione peroxidase (GPX), genome-wide, stress, gene expression

## Abstract

Glutathione peroxidase (GPX) is crucial in mediating plant responses to abiotic stresses. In this study, bioinformatics methods were used to identify the *GPX* gene family in quinoa. A total of 15 *CqGPX* genes were identified at the quinoa genome level and conducted preliminary analysis on their protein characteristics, chromosome distribution, gene structure, conserved domain structure, cis-acting elements, and expression patterns. Phylogenetic analysis showed that the *GPX* genes of quinoa, *Arabidopsis*, soybean, rice, and maize were divided into three groups. Most of the CqGPXs had the three characteristic conserved motifs and other conserved sequences and amino acid residues. Six types of cis-acting elements were identified in the *CqGPX* gene promoter, with stress and hormone response-related cis-acting elements constituting the two main categories. Additionally, the expression patterns of *CqGPX* genes across various tissues and their responses to treatments with NaCl, PEG, CdCl_2_, and H_2_O_2_ were also investigated. The qRT-PCR results showed significant differences in the expression levels of the *CqGPX* genes under stress treatment at different time points. Consistently, the activity of glutathione peroxidase enzymes increased under stresses. Heterologous expression of *CqGPX4* and *CqGPX15* conferred stress tolerance to *E. coli*. This study will provide a reference for exploring the function of *CqGPX* genes.

## 1. Introduction

Abiotic stress refers to environmental conditions that are not conducive to the survival, growth, and development of plants, and even lead to injury, destruction, and death. During plant growth and development, there are abiotic stresses including salinity, drought, excessive light, and hypoxia [1,2,3]. Abiotic stress induces plants to produce and accumulate excessive reactive oxygen species (ROS), which causes oxidative stress and triggers non-functional modification of nucleic acids, lipids, and proteins, resulting in defects in cell function and seriously affecting their growth and development [4,5,6]. In order to adapt to various environmental stresses and resist damage caused by excessive ROS, plants have an antioxidant system mainly based on enzymatic antioxidant mechanisms, among which the main enzymes include catalase (CAT), ascorbate peroxidase (APX), and glutathione peroxidase (GPX) [7,8].

GPX (EC 1.11.1.9) is a non-heme peroxidase that uses glutathione (Glutathione, GSH) or thioredoxin (Thioredoxin Reductase, TRX) as a reductant to catalyze the reduction of hydrogen peroxide (H_2_O_2_), lipid peroxides, and organic hydroperoxides to water or corresponding alcohols [9,10]. GPX in mammals uses GSH as electron donor, and the activity of animal Sec-GPX is 2–3 orders of magnitude higher than that of Cys-GPX. The catalytic active site of plant GPX contains cysteine residue rather than selenocysteine. Current studies show that some plant GPX has strong activity only when TRX is used as electron donor, so plant GPX is essentially a peroxidase dependent on TRX [11,12,13,14]. GPX is widely found in different tissues, organs and cells of plants. It exists in the nucleus, chloroplast, mitochondria, cytoplasm, Golgi apparatus, and endoplasmic reticulum—a distribution pattern consistent with ROS generation throughout cellular compartments [15,16].

GPX is a crucial component of the enzymatic antioxidant system. It not only clears excess ROS produced within cells but also regulates ROS concentration to transmit signals, playing an important role in plant growth and development. It is particularly vital for plants to resist abiotic stresses such as drought, high temperatures, salt stress, and heavy metal toxicity, as well as biotic stresses [17]. Eight gene families homologous to mammalian glutathione peroxidase (GPX) isoenzymes have been identified in *A. thaliana*, most of which were up-regulated coordinately in response to abiotic stresses [18,19]. By analyzing the T-DNA insertion mutation lines and overexpression lines, the roles of *Arabidopsis GPX* genes (*AtGPX1*-*AtGPX8*) were systematically studied [20,21,22,23]. The T-DNA insertion mutant *atgpx3* exhibited sensitivity to drought and H_2_O_2_ stress and increased the production of H_2_O_2_ in guard cells. *ATGPX3* might sense and transduce the oxidative signal by interacting with 2C type protein phosphatase abscisic acid insensitive 1 and 2 (ABI1 and 2) in ABA and drought stress signaling [21]. Constitutive overexpression of *AtGPX5* lead to the improvement of salt tolerance in *Arabidopsis* [22]. Compared with wild-type plants, the knockout *Arabidopsis* mutant of *AtGPX8* showed higher sensitivity to oxidative damage during root elongation. In contrast, lines engineered to overexpress *AtGPX8* had a higher tolerance to oxidative damage than wild-type plants [19,23]. The binding mode and molecular interaction mechanism between *Arabidopsis* glutathione peroxidase AtGPX6 and copper ions contribute to elucidating the damage mechanism of heavy metal exposure to antioxidant defense systems at the molecular level [24]. The depletion of *GPX1* and *GPX7* expression in *Arabidopsis* chloroplasts leads to impaired tolerance to photooxidative stress, increased basal resistance to virulent bacteria, and severe morphological changes in leaf cells and chloroplasts [25]. In rice, the mitochondrial *GPX1* gene silencing lines exhibit growth reduction, photosynthesis impairment, and greater sensitive to salinity stress [26]. The mitochondrial glutathione peroxidase OsGPX3 modulates shoot and root development and H_2_O_2_ homeostasis in rice [27]. Overexpression of *NaGPX* from *Nelumbo nucifera* enhances salt stress tolerance in rice [28]. Overexpression of wheat *GPX* genes *W69* and *W106* enhanced *Arabidopsis*’ tolerance to salt stress, H_2_O_2,_ and ABA treatments [29]. All these findings indicated that *CqGPXs* genes in different plant species could regulate plant development processes, stress responses, and more.

Quinoa (*Chenopodium quinoa* Willd.) is native to the Andes Mountains of South America, where the climate is harsh, and the living environment of drought, high altitude, frost, and more, accompanied by cold damage and poor soil, gives quinoa the characteristics of salt tolerance, cold hardiness, drought adaptation, UV-B radiation resilience, and other resistance to abiotic stresses. This study aims to screen and identify members of the *CqGPXs* gene family in quinoa using bioinformatics methods and conduct systematic analysis of the *CqGPXs* gene family, including physicochemical properties, subcellular localization, gene structure, conserved domains, and expression profiles. Additionally, *CqGPX4* and *CqGPX15* were functionally characterized in *E. coli*.

## 2. Materials and Methods

### 2.1. Identification of the CqGPXs Gene Family in Quinoa

In this study, the candidate *CqGPX* genes in quinoa were identified using BLASTP and the HMMER tool [30]. A total of 15 AtGPX protein sequences were downloaded from the *Arabidopsis* website (https://www.arabidopsis.org/, accessed on 7 June 2024). Quinoa genome sequence downloaded from the data (http://www.cbrc.kaust.edu.sa/chenopodiumdb/, accessed on 27 August 2024)). The retrieval threshold was set as E-value < E^−10^. To further select *CqGPX* genes, the NCBI-CDD databases (https://www.ncbi.nlm.nih.gov/cdd/, accessed on 2 September 2024) and Pfam (http://pfam.xfam.org/search tabview, accessed on 2 September 2024) were used for checking the presence of the GPX domain (registration numbers PF00255). The physicochemical characteristics were determined using ExPASy (https://web.expasy.org/protparam/, accessed on 7 June 2024). The WoLF PSORT Ⅱ online software (http://www.genscript.com/wolf-psort.html, accessed on 7 June 2024) [31] was used to predict the subcellular localization of *CqGPXs*.

### 2.2. Evolutionary Relationship of the CqGPXs Gene Family

Quinoa CqGPX protein sequences and *Glycine max* (GmGPX), *Arabidopsis thaliana* (AtGPX), *Oryza sativa* (OsGPX), and *Zea mays* (ZmGPX) protein sequences were used to construct the phylogenetic tree. MEGA 7 software (v7.0.26) was used to align multiple protein sequences, and the neighbor-joining (NJ) scheme was executed to produce the final comparison results with 1000 bootstrap replicates. We used the iTOL (https://itol.embl.de/, accessed on 5 September 2024) platform to visualize and refine the generated phylogenetic tree [15].

### 2.3. Gene Structure and Protein Conserved Motif Analysis

We extracted gene structures from genome annotation gff3 files and visualized them using TBtools v2.067. MEME (http://meme-suite.org/tools/meme, accessed on 7 September 2024) [32] was used to identify the conserved motifs of CqGPXs. DNAMAN v.8.0 software conducted multiplex sequence alignment of CqGPXs amino acid sequences [33].

### 2.4. Chromosomal Location and Gene Duplication Analysis

Within the quinoa genome, genomic synteny analysis was carried out using Tbtools-II v2.025. All-against-all BLASTP alignments were conducted to pinpoint homologous gene pairs [34]. The MCScanX plugin, which is incorporated into TBtools, was utilized to identify segmental duplications and syntenic blocks. TBtools was then used to visualize the duplication events and synteny outcomes. Moreover, TBtools was employed to compute the synonymous (Ks) and nonsynonymous (Ka) substitution rates for homologous quinoa gene pairs.

### 2.5. Analysis of Cis-Acting Elements in the Promoter Regions

The promoter sequence (2000 bp upstream region from translation start code ATG of the putative genes) was obtained by TBtools, and the cis-acting elements within it were predicted using the PlantCARE database (http://bioinformatics.psb.ugent.be/webtools/plantcare/html/, accessed on 15 September 2024) [35].

### 2.6. Analysis of CqGPXs Gene Expression Patterns in Diverse Tissues

To study the expression of *CqGPX* genes in different tissues and organs, we downloaded the quinoa RNA-seq data (SRP226463, SRP116149, SRX1967556, SRX1967551) from the SRA database (http://www.ncbi.nlm.nih.gov/sra, accessed on 21 September 2024). RNA-seq data were standardized in the form of TPM (transcripts per million reads), and we performed log2 conversion. TBtools software was used to visualize the heatmap of *CqGPXs* gene expression.

### 2.7. Plant Materials and Stress Treatments

Quinoa YT077 was used as the experimental material. Quinoa was grown in a greenhouse with controllable environmental conditions: 22 °C, 70–75% relative humidity, and 16 h of light and 8 h of dark photoperiod. One-month-old quinoa seedlings exhibiting consistent growth were selected for exposure to 300 mM NaCl (salt stress), 20% PEG6000 (drought stress), 500 µM CdCl_2_, and 10 mM H_2_O_2_. The leaves and roots of seedlings were taken at 0 h, 3 h, 6 h, 12 h, 24 h, and 48 h after treatment. The collected samples were frozen quickly with liquid nitrogen and placed at −80 °C until further usage. Three biological replicates for each treatment [36].

### 2.8. RNA Extraction and qRT-PCR Analysis

TransZol Up Plus RNA Kit (No. ER501, TransGen Biotech, Beijing, China) was used to extract total RNA from plant materials, and the cDNA synthesis was performed using TransScript^®^ One-Step gDNA Removal and cDNA Synthesis SuperMix (No. AT311, TransGen Biotech, Beijing, China). The qRT-PCR was conducted with QIAGEN Rotor-Gene Q (Hilden, Germany) using TransStart^®^ Top Green qPCR SuperMix (No. AQ131, TransGen Biotech, Beijing, China). The quinoa *Tubulin* (*CqTub*) gene was used as an internal control. All the primers utilized in this experiment are listed in Table S5. The reaction conditions were set as follows: pre-denaturation at 95 °C for 1 min; 40 cycles of 95 °C for 10 s, 58 °C for 10 s, and 72 °C for 20 s. The 2^−∆∆CT^ method was used to calculate the relative expression of genes.

### 2.9. Enzyme Activity Mensuration

One-month-old quinoa seedlings were subjected to 4 stresses, and their leaves were harvested at 24 h and 48 h post-stress to measure enzyme activity. We used the glutathione peroxidase activity assay kit (code BC1195, Beijing, China) to measure GPX activity in quinoa leaves and calculated enzyme activity using the formula provided in the kit manual [37].

### 2.10. Heterologous Expression of CqGPX4 and CqGPX15 in E. coli and Stress Tolerance Assay

The primers (Table S5) for the cloning of *CqGPX4* and *CqGPX15* were designed using the Primer 3. These two genes were amplified from the quinoa cDNA, then cloned into the protein expression vector pET28a. The recombinant plasmids were sequenced for validation and transformed into *E. coli* BL21 (DE3) strain. The seed solution of *E. coli* BL21 (DE3) was transferred to 50 mL/250 mL LB medium (50 μg/mL kanamycin) and incubated at 37 °C, 180 r/min to an OD600 of about 0.6–0.8, and IPTG (final concentration of 1 mM) was added to induce expression to the logarithmic phase. After low-temperature induction, centrifugation was used to collect the bacterial solution and it was rinsed with PBS. To verify the successful induction of the target protein, we conducted a 12% SDS-PAGE with Coomassie brilliant blue staining.

To compare the effects of two *CqGPXs* genes on the growth of *E. coli* under stress, *E. coli* strains containing pET28a::*CqGPX4*, pET28a::*CqGPX15*, or pET28a (as a negative control) were cultured in LB liquid medium with or without 400 mM NaCl, 800 mM PEG, 500 µM CdCl_2_, and 2 mM H_2_O_2_ (50 µg/mL kanamycin + 1 mM IPTG) at 28 °C and 180 r/min. The OD600 was assayed at two-hour intervals. In addition, these *E. coli* strains containing pET28a::*CqGPX4*, pET28a::*CqGPX15*, or pET28a were also cultured on LB agar plates containing 400 mM NaCl, 500 mM mannitol, 500 µM CdCl_2_, and 0.4 mM H_2_O_2_ (50 µg/mL kanamycin + 1 mM IPTG) for 24 h. We observed the growth status and took photos to record it.

## 3. Results

### 3.1. Identification of CqGPX Genes in Quinoa

Through BLASTP and HMM search, a total of 18 candidate *GPX* genes were identified in quinoa (*Chenopodium quinoa*). Analysis with Pfam and CDD programs revealed that 3 of the 18 candidate GPX proteins lacked the GPX protein family domain (PF00255). Therefore, 15 genes were identified as members of the *CqGPXs* family and renamed as *CqGPX1* to *CqGPX15* based on their order on chromosomes (Table 1). The proteins encoded by the quinoa *CqGPXs* genes contains 141–455 amino acid residues, with a molecular weight ranging from 15.98 to 50.87 KDa. The isoelectric point span is 4.54–9.43, indicating the presence of both acidic and alkaline proteins. Most CqGPXs are hydrophilic stable proteins. One CqGPX protein is located in the nucleus, four CqGPX proteins are located in the mitochondria, and the remaining ten CqGPX proteins are located in chloroplasts or mitochondria.

### 3.2. Phylogenetic Analysis of CqGPX Genes

**Figure 1:**
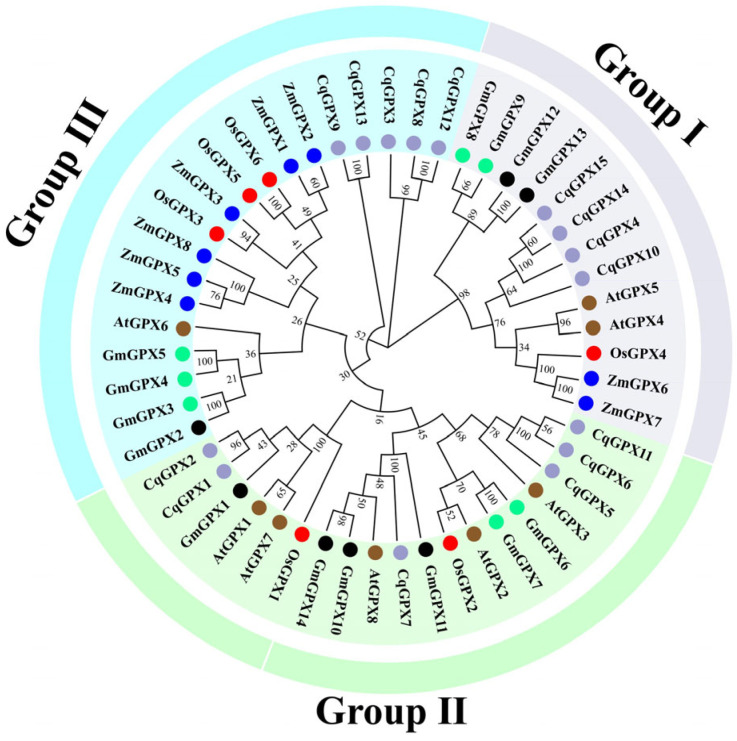
Phylogenetic relationship analysis of GPXs from *C. quinoa* (CqGPX), *Zea mays* (ZmGPX), *Oryza sativa* (OsGPX), soybean (GmGPX), and *A. thaliana* (AtGPX).

To investigate the phylogenetic relationships of *CqGPX* genes, a maximum likelihood phylogenetic tree was constructed based on the alignment of GPX protein sequences from *Arabidopsis thaliana* (8 AtGPXs), *Glycine max* (14 GmGPXs), *Oryza sativa* (6 OsGPXs), *Zea mays* (8 ZmGPXs), and *C. quinoa* (15 CqGPXs) (Table S1). The results indicated that these proteins were classified into three groups based on sequence similarity, namely Group I, Group II, and Group III. Group I consisted of 13 GPX members, including 4 quinoa GPX proteins (CqGPX4, CqGPX10, CqGPX14, and CqGPX15); Group II consisted of 19 GPX members, including 6 quinoa GPX proteins (CqGPX1, CqGPX2, CqGPX5, CqGPX6, CqGPX7, and CqGPX11); Group III consisted of 19 GPX members, including 5 quinoa GPX proteins (CqGPX3, CqGPX8, CqGPX9, CqGPX12, and CqGPX13). It was noteworthy that the sequences of CqGPX were more closely related to *Arabidopsis* GPX (AtGPX) and soybean GPXs (GmGPX) than to maize GPX (ZmGPX) and rice GPX (OsGPX) (Figure 1).

### 3.3. Gene Structural and Conserved Domain Analyses of CqGPXs

**Figure 2:**
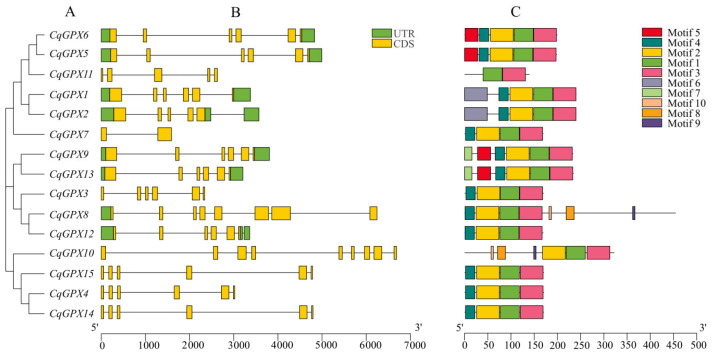
Gene structure and conserved protein motifs in CqGPXs. (**A**) A phylogenetic tree of the *CqGPX* gene family. (**B**) Exon-intron distribution of CqGPXs. The UTR regions, exons, and introns are represented with green boxes, yellow boxes, and black lines, respectively. (**C**) The conserved motif of CqGPXs. Each motif is presented in a specific color.

Different genes have varying numbers of introns and exons. In quinoa, *CqGPX* genes contain a range of introns from 1 to 8 and exons from 2 to 9 (Figure 2B). *CqGPX1*, *CqGPX3*, *CqGPX4*, *CqGPX5*, *CqGPX6*, *CqGPX9*, *CqGPX13*, *CqGPX14*, and *CqGPX15* all contain 6 exons and 5 introns. *CqGPX7* contains 2 exons and 1 intron. *CqGPX8* contains 8 exons and 7 introns. *CqGPX10* contains 9 exons and 8 introns. *CqGPX11* contains 5 exons and 4 introns. *CqGPX2* contains 5 exons and 5 introns. *CqGPX12* contains 6 exons and 6 introns. We found an intron in the 3′UTR region of *CqGPX2* and *CqGPX12*.

The 15 CqGPXs proteins contain 10 conserved motifs (Table S2). As illustrated in (Figure 2C), the number of conserved motifs in CqGPX proteins ranged from 2 to 7. Among them, 15 CqGPX proteins all contained motif 1 and motif 3, which carried the GPX signature. Except for CqGPX11, most of them possessed motif 2, and except for CqGPX10 and CqGPX11, most of them possessed motif 4. Besides this, CqGPX5, CqGPX6, CqGPX9, and CqGPX13 carried an additional motif 5, and CqGPX1 and CqGPX2 carried an additional motif 6. The multiple alignments results of the 15 CqGPXs showed that most of CqGPX members contained three highly conserved motifs except for CqGPX11. These three motifs are called GPX signature 1 (GKVLLIVNVASXCG), GPX signature 2 (ILAFPCNQ), and GPX signature 3 (WNFXKF). CqGPX11 did not contain GPX signature 1. Additionally, we discovered that each of them possesses one or more highly conserved potential catalytic residues: Cys (C), Gln (Q), Trp (W), and Asn (N) (Figure S1). These motif differences might be the potential basis for functional differences between various CqGPXs.

### 3.4. Chromosomal Location and Duplication of CqGPX Genes

**Figure 3:**
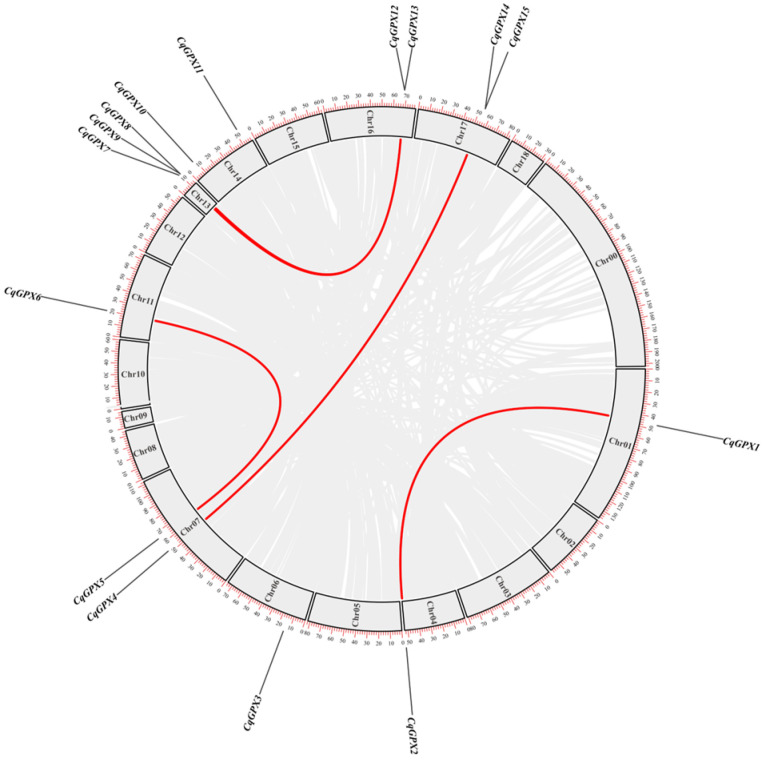
Chromosomal distribution and gene duplication of *CqGPX* genes. The red line represents the duplicate pairs of *GPXs* genes in the quinoa genome, while all homologous blocks are represented by gray lines. Chr00-Chr18 chromosome numbers represent each chromosome.

The 15 *CqGPX* genes are unevenly distributed on chromosomes 1, 4, 6, 7, 11, 13, 14, 16, and 17. Five gene pairs were identified through gene duplication analysis based on *CqGPXs* sequence similarity, and segmental duplication was observed in all five gene pairs (Figure 3). These findings indicated that segmental duplication plays an important role in promoting the expansion of the *CqGPXs* genes in quinoa. We also calculated the Ka/Ks ratios of all gene duplicates to investigate the evolutionary selection of the *CqGPX* gene family (Table S3). The results indicated that the values were consistently below 1, suggesting that purification selective pressure was the main evolutionary pressure for *CqGPX* genes.

### 3.5. Cis-Acting Elements Analysis

**Figure 4:**
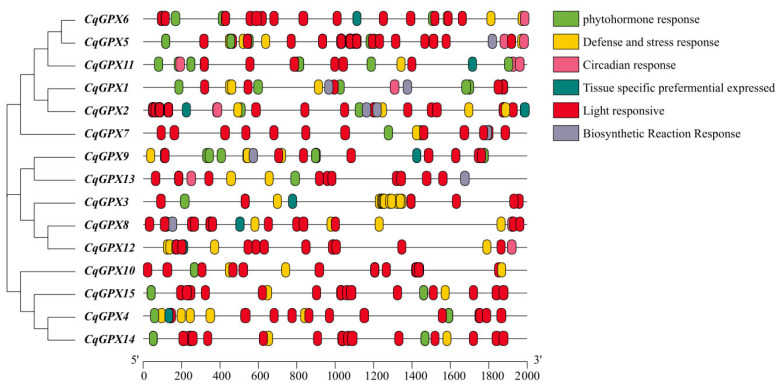
Cis-acting elements in the promoter of *CqGPX* genes.

In order to gain a deeper understanding of the precise regulation of the *CqGPX* genes, we extracted and analyzed a 2000 bp sequence located upstream of the start codon of the *CqGPX* genes and identified the cis-element using the PlantCARE tool (Figure 4). Within the 2000 bp promoter range, different *CqGPX* genes contain different types of response elements. In the promoter regions of *CqGPXs*, we identified six major types of cis-acting elements related to circadian response, defense and stress response, light response, phytohormone response, tissue-specific preferential expressed, and biosynthetic reaction response (Table S4). Each *CqGPX* gene contains 4–10 functional elements. The phytohormone response elements included those responding to auxin (TGA-elements), gibberellins (TATC-box and P-box, and GARE-motif), salicylic acid (TCA-element), abscisic acid (ABRE), and MeJA response (TGACG-motif and CGTCA-motif). The defense and stress response elements included those involved in defense response (TC-rich repeats), low-temperature response (LTR), anoxic response (ARE and GC motif), and drought-inducibility (MYB). These analysis results indicated that the *CqGPX* genes might be widely involved in mediating responses to hormones and stress.

### 3.6. Analysis of CqGPXs Expression Patterns in Tissues

**Figure 5:**
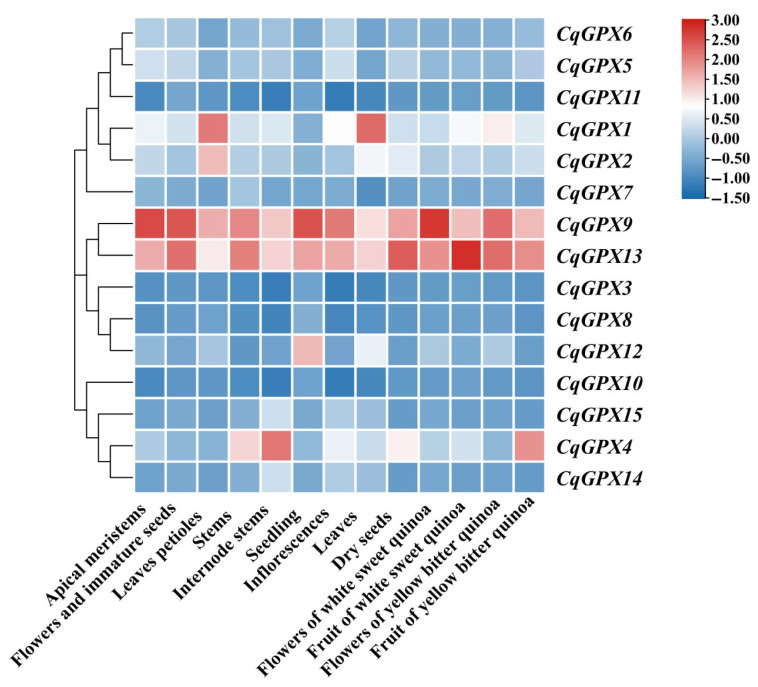
Expression profiles of *CqGPXs* genes in different quinoa tissues.

To investigate the potential role of *CqGPX* genes in quinoa development, their expression profiles across multiple tissues were analyzed by searching and reanalyzing RNA-Seq data from the NCBI SRA database. *CqGPX9* and *CqGPX13* showed high expression patterns in 13 different tissues, indicating that they may play important roles in the development of quinoa (Figure 5). *CqGPX1* showed specific and high expression levels in leaves and leaves petioles, implying their specific roles in leaf development. *CqGPX4* exhibited the highest expression level in internode stems and relatively higher expression in stems, indicating that it may play specific roles in stem development. Also, the expression level of *CqGPX4* was high in the fruit of yellow bitter quinoa. *CqGPX2* showed specific and relatively high expression levels in leaf petioles and *CqGPX12* was specifically expressed in seedlings. These results indicate that there are different tissue-specific expression patterns among *CqGPX* genes.

### 3.7. Analysis of CqGPX Genes Expression Patterns Under Stress Conditions

**Figure 6:**
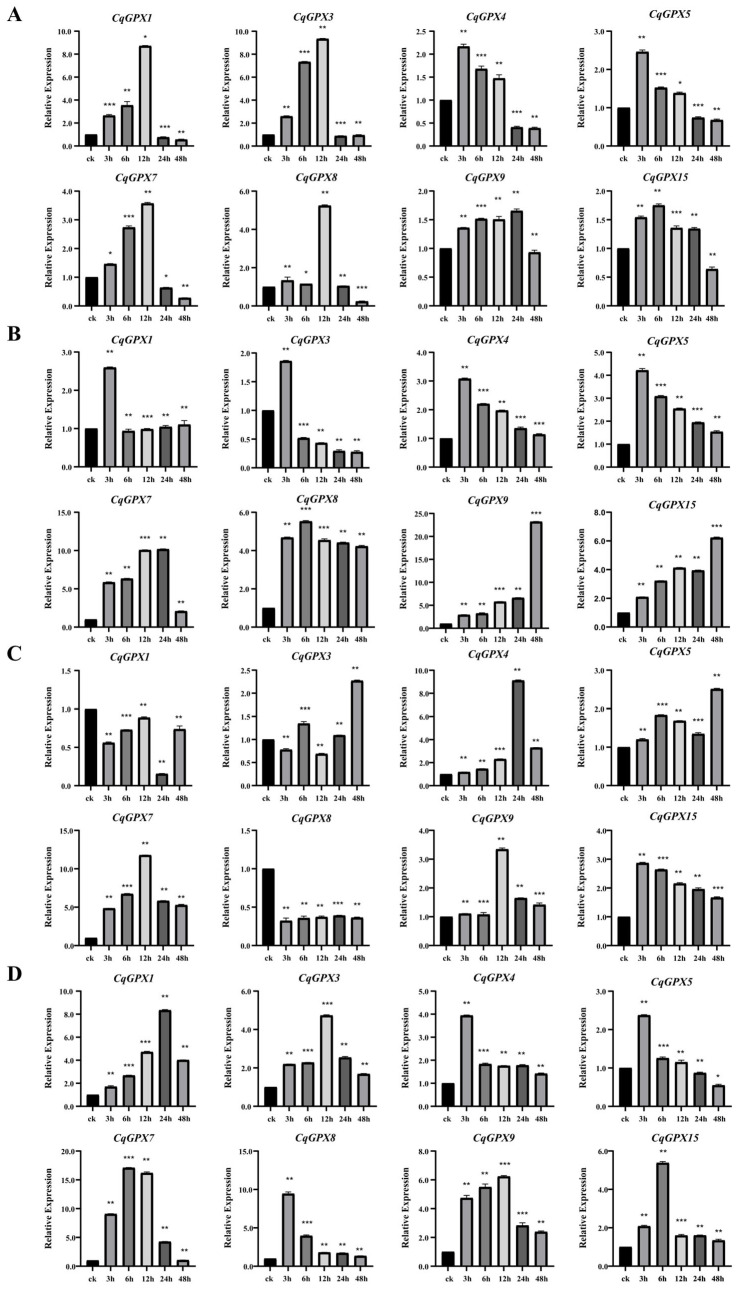
Expression analyses of *CqGPX* genes via qRT-PCR under various stress conditions, including (**A**) 300 mM NaCl, (**B**) 20% PEG 6000, (**C**) 500 µM CdCl_2_, and (**D**) 10 mM H_2_O_2_. Student’s *t*-test: * *p* < 0.05; ** *p* < 0.01; *** *p* < 0.001.

In this study, we employed qRT-PCR to analyze the expression profiles of eight *CqGPX* genes under different conditions (NaCl, PEG, CdCl_2_, and H_2_O_2_) (Figure 6). Under NaCl stress, all eight genes, especially *CqGPX1* and *CqGPX3*, were significantly induced, and the expression peaks reached 8-fold and 9-fold of the control level. Specifically, the expression level of *CqGPX1*, *CqGPX3*, *CqGPX7*, and *CqGPX8* gene increased first and then decreased with the increase in treatment time, reaching a peak at 12 h. The expression patterns of *CqGPX4* and *CqGPX5* were similar, reaching the highest point at 3 h of treatment, about twice that of the control, and then slowly decreasing. Although the expression levels of *CqGPX9* and *CqGPX15* were not statistically significant relative to other genes, both exhibited an initial increase followed by a decrease, peaking at 6 h with approximately 1.8-fold higher expression than the control group. Under PEG stress, all eight genes, especially *CqGPX7* and *CqGPX9*, were significantly induced, and the expression peaks reached 10-fold and 20-fold that of the control, respectively. Specifically, the expression levels of *CqGPX4* and *CqGPX5* gene first increased and then decreased during treatment and surged to their peak at 3 h, which was three times that of the control group, and then decreased steadily with time, but the expression levels were still higher than that of the control group. The expression of *CqGPX15* increased continuously until 48 h, and the expression level was about 6 times that of the control group. Under CdCl_2_ treatment, the expression of *CqGPX3*, *CqGPX4*, *CqGPX5*, *CqGPX7*, *CqGPX9*, and *CqGPX15* were up-regulated, reaching their maximum values at 48 h, 24 h, 48 h, 12 h, 12 h, and 3 h, respectively. However, *CqGPX8* was significantly down-regulated under CdCl_2_ treatment. The expression of all *CqGPX* genes was up-regulated at certain time points after H_2_O_2_ treatment. The expression of *CqGPX4*, *CqGPX5*, and *CqGPX8* significantly increased after H_2_O_2_ treatment, reached its peak at 3 h, and then decreased. This suggests that these genes may play an important role in the early stages of responding to oxidative stress. In summary, the expression patterns of *CqGPX* genes indicated that these genes were responsive to stress.

### 3.8. Determination of the Activity of CqGPX Enzyme Under Stress Conditions

**Figure 7:**
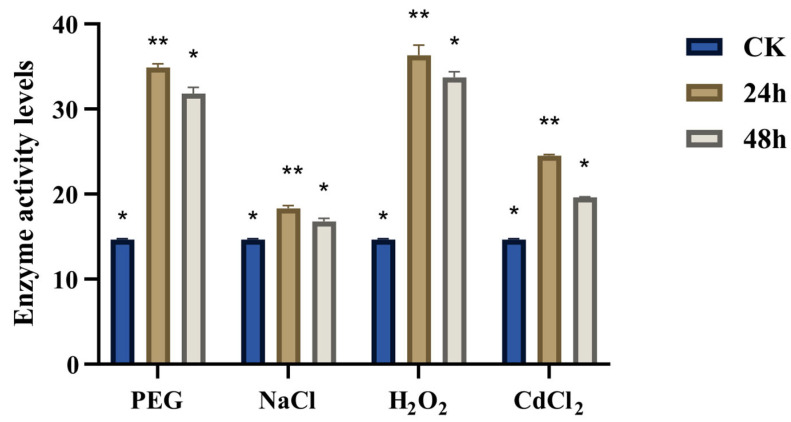
Analysis of glutathione peroxidase activity under 300 mM NaCl, 20% PEG 6000, 10 mM H_2_O_2_, and 500 µM CdCl_2_ treatments. Statistically significant differences are indicated by * *p* < 0.05, ** *p* < 0.01, using one-way ANOVA.

Considering that the expression of most *CqGPX* genes was significantly regulated in quinoa under PEG, NaCl, H_2_O_2_, and CdCl_2_ stress conditions, we assayed GPX activity enzyme under these stress conditions to confirm their functional relationship with quinoa stress tolerance. As shown in Figure 7, after 24 and 48 h of treatment with PEG and H_2_O_2_, the GPX activity in quinoa tissue significantly increased by about 2 fold. However, after 24 h of treatment with CdCl_2_, the activity increased by about 1.5 times. Under salt stress, the activity slightly increased at 24 and 48 h.

### 3.9. Overexpression of CqGPX4 and CqGPX15 Enhanced Stress Tolerance in E. coli

**Figure 8:**
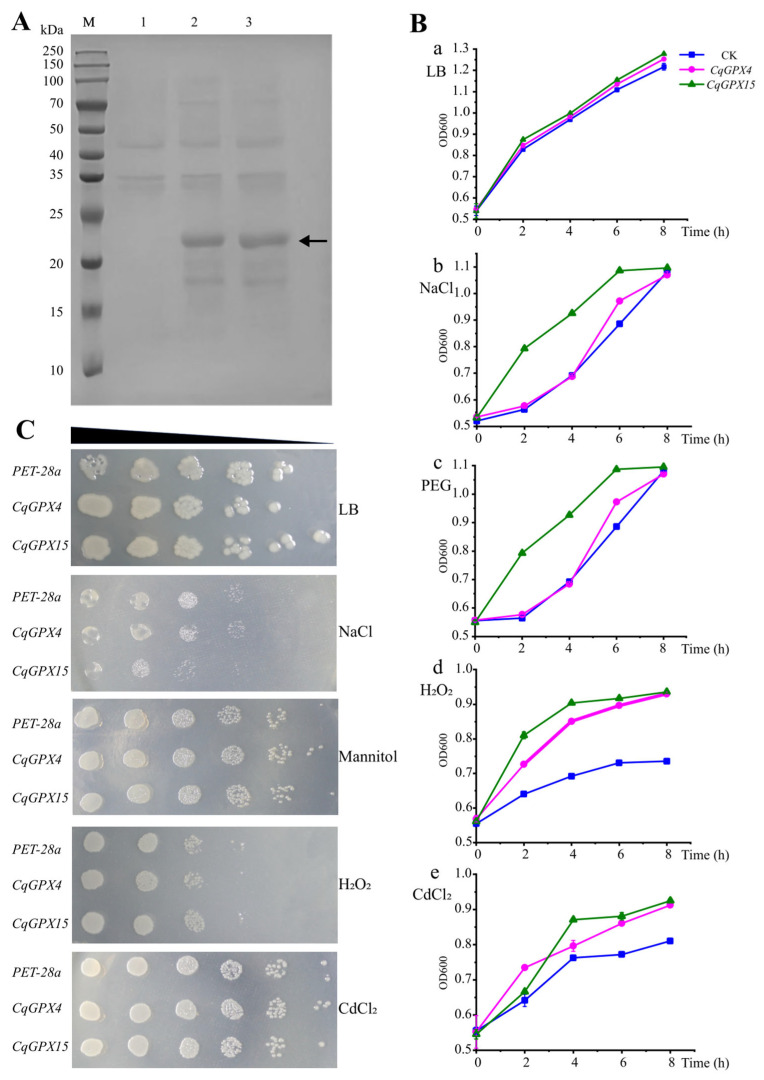
Stress resistance analysis of heterologous expression of *CqGPX4* and *CqGPX15* in *E. coli* strain BL21. (**A**) Expression analysis of the recombinant protein in *E. coli*. The whole cell lysates were separated by SDS-PAGE. Lane M: protein marker; lane 1: induced BL21 (pET28a); lane 2: induced BL21 (pET28a::*CqGPX4*); lane 3: induced BL21 (pET28a::*CqGPX15*). (**B**) The growth curves of BL21/pET28a, BL21/pET28a::*CqGPX4*, and BL21/pET28a::*CqGPX15* under stress conditions. The recombinant *E. coli* strain BL21 were cultivated in LB medium or LB medium supplemented with 400 mM NaCl, 700 mM PEG, 2 mM H_2_O_2_, and 500 µM CdCl_2_ for comparison. Data represent the mean and standard deviation of three independent experiments. (**C**) Spot assay showing better growth of recombinant BL21/pET28a::*CqGPX4*, and BL21/pET28a::*CqGPX15* under various stress conditions (400 mM NaCl, 500 mM mannitol, 0.4 mM H_2_O_2_, and 500 µM CdCl_2_) up to 10^5^-fold dilutions when compared with BL21/pET28a.

To verify the role of *CqGPXs* under different stresses, we constructed prokaryotic expression vectors for *CqGPX4* and *CqGPX15* and transformed them into *E. coli* BL21 (DE3) strain. The successful expression of CqGPX4 and CqGPX15 proteins in *E. coli* was detected using 12% SDS-PAGE staining with Coomassie Brilliant Blue R-250 (Figure 8A). Under fresh LB liquid culture conditions, there was no significant difference in the growth curve of cells transformed with empty vectors or recombinant plasmids. However, in LB liquid medium supplemented with 400 mM NaCl, 700 mM PEG, 2 mM H_2_O_2_, and 500 µM CdCl_2_, the BL21 (DE3) strain carrying *CqGPX4* and *CqGPX15* grew better than the native control (empty vector), the growth kinetics of the BL21 (DE3) strain carrying *CqGPX4* and *CqGPX15* were superior to those with empty vector under PEG and NaCl treatment, and even better growth was observed under H_2_O_2_ and CdCl_2_ treatment (Figure 8B). In addition, the pattern of BL21 (DE3) growth was also tested on solid medium supplemented with 400 mM NaCl, 500 mM mannitol, 0.4 mM H_2_O_2_, and 500 µM CdCl_2_ (Figure 8C). The BL21 (DE3) strain carrying *CqGPX4* and *CqGPX15* showed higher growth rate than those carrying empty vector. These results indicated that overexpression of *CqGPX4* and *CqGPX15* increased the tolerance of *E. coli* strains to salinity, osmotic stress, Cd^2+^ stress, and oxidative stress.

## 4. Discussion

When plants are subjected to abiotic and/or biotic stress, ROS is often overproduced, which can lead to oxidative stress and damage to DNA, lipids, and proteins, thereby impacting plant growth and development. Glutathione peroxidase (GPX) effectively eliminates ROS, thereby reducing the damage in plants [38]. The *GPX* gene families have been successfully identified in various plants, including five *CaGPX* genes in *Capsicum annuum* [39] and in *Cicer arietinum* [40], five *PdGPX* genes in *Phoenix dactylifera* [41], six *ClGPX* genes in *Citrullus lanatus* [42], six *CsGPX* genes in *Cucumis sativus* [43], eight *AtGPX* and *TsGPX* genes in *Arabidopsis thaliana* and *Thellungiella salsuginea* [44], thirteen *GmGPX* genes in *Glycine max*, thirteen *GsGPX* gene*s* in *Glycine soja* [45], fourteen *NtGPX* genes in *Nicotiana tabacum* [46], and twenty-five *BnGPX* genes in *Brassica napus* [15]. In this study, we identified 15 *CqGPX* genes in quinoa. The differences in the number of *GPX* genes among different plant species may be attributed to gene duplication and whole genome duplication (WGD), including tandem and segmental duplication. There were two and eight segmental duplication events in *Arabidopsis* and rapeseed, respectively [15,47]. We found that *CqGPXs* underwent segmental duplication and purifying selection during their evolution, which was similar to other gene families in quinoa such as *CqACSs* and *CqPP2Cs* [48,49].

In the current study, subcellular localization analysis indicated that CqGPXs were mainly distributed in chloroplasts and/or mitochondria, while CqGPX10 was localized in the nucleus. Plant chloroplasts and mitochondria contain high levels of ROS due to light reactions and respiration, respectively. The GPX enzymes present in these organelles can timely and efficiently eliminate ROS, protecting organelles from oxidative damage [10]. Similar discoveries have also been described in *Arabidopsis* [16,25], rapeseed [15], tobacco [46], and pepper [39]. Of course, the subcellular localization of CqGPXs in quinoa still needs to be further studied through experimental methods.

The phylogenetic tree indicates that the *CqGPX* genes of quinoa and four other plants are categorized into three primary groups (Figure 1), which is consistent with the grouping of other plants such as chickpea and wheat [40,50]. Conservative motif analysis (Figure 2) and multiple sequence alignment (Figure S1) indicate that, except for CqGPX11, the other 14 CqGPXs have three highly conserved motifs (motifs 1, 2, and 3), including three GPX signatures. CqGPX11 is missing motif 2 (GPX signature 1). The GPX signature 1 (GKVLLIVNVASXCG) is the catalytic activity region of plant GPXs. In tobacco, NtGPX3 subfamily members also lack motifs (GPX features) [46]. Furthermore, some evolutionarily conserved residues were also identified in CqGPX proteins, including Cys (C), Gln (Q), Trp (W), and Asn (N) (Figure S1). Unlike animals, the enzymatic activity of plant GPXs relies on the three crucial cysteine residues (Cys) [12,16]. Mutations in the three conserved Cys residues within the active site of PgGPX resulted in complete loss of activity [51]. However, in CqGPX3 and CqGPX11, there are substitutions or deletions of these three crucial cysteine residues. Similarly, in potato StGPXL2, StGPXL4, and StGPXL5 and watermelon ClGPX1 and ClGPX4, these three key cysteine residues also have substitutions and deletions [42,52]. All these results indicate that CqGPX3 and CqGPX11 are similar to NtGPX3, StGPXL2, StGPXL4, StGPXL5, ClGPX1, and ClGPX4, may lose or acquire new particular functions, and may have unique evolutionary trajectories.

Through analysis of cis-elements, it was found that the tissue specific preferential expressed element in some *CqGPX* gene promoter regions (Figure 4, Table S4). The expression patterns of *CqGPX* genes in 13 quinoa tissues were studied using RNA-seq data from NCBI database (Figure 5), indicating the existence of different tissue-specific expression patterns among *CqGPX* genes. Many studies have shown that the *GPX* genes exhibit tissue-specific expression patterns. For example, six tobacco *NtGPX* genes showed high expression in mature leaves and four tobacco *NtGPX* genes showed high expression in stems [46]. In all tissues of rapeseed, most groupⅡgenes showed high expression [15]. In *L. japonicus*, *LjGPX1* and *LjGPX3* are highly expressed in leaves, roots, and nodules [53].

Under various stress conditions, *GPX* gene expression was up-regulated to enhance plant stress tolerance. For example, the *AtGPX* expression levels were significantly up-regulated by exogenous NaCl, mannitol, cold, heat, FeSO_4_, or CuSO_4_ treatment [18]. In *Pennisetum glaucum*, the *PgGPX* expression levels were also significantly up-regulated in response towards salt and drought stresses [51]. In *Glycine soja*, most *GsGPX* genes showed an increase in expression in response to H_2_O_2_ treatment [45]. In *Brassica napus*, *BnGPX21* and *BnGPX23* were significantly up-regulated under waterlogging, cold, salt, and drought stress [15]. Our cis-element analysis results indicated the presence of numerous stress and hormone responsive cis-elements in the *CqGPXs* promoter (Figure 4, Table S4), suggesting that the *CqGPX* genes may be involved in multiple stress and hormone responses. In current study, we analyzed the expression profiles of eight *CqGPX* genes under different environmental conditions by qRT-PCR. We found that nearly all *CqGPX* genes were significantly up-regulated under NaCl, PEG, CdCl_2_, and H_2_O_2_ treatments, which is consistent with the results of other plant species. However, we found that *CqGPX8* was significantly down-regulated under CdCl_2_ treatment. In tobacco and common carp, most *NtGPX* and *CcGPX* genes were down-regulated during the initial CdCl_2_ treatment stage [46,54].

Several investigations have shown that increased GPX activity under various stresses. Exogenous 24-epi-brassinolide (EBR) increased glutathione peroxidase activity under low temperature stress in the cold-sensitive tomato varieties [55]. Salicylic acid pretreatment could enhance GPX activity and significantly reduce the negative effects of salt stress on tomatoes [56]. Short term cadmium treatment could rapidly stimulate the expression and activity of GPX in barley roots [57]. Under ABA, cold, PEG, and NaCl treatment, the activity of glutathione peroxidase in pepper first increased and then decreased [58]. In our study, we found that the activity of glutathione peroxidase in quinoa was significantly increased under treatment with NaCl, PEG, CdCl_2_, and H_2_O_2_.

Moreover, the phylogenetic tree results indicated that *CqGPX4* and *CqGPX15* belong to Group I along with *OsGPX4*, *AtGPX4*, and *AtGPX5*. Previous research results had shown that *OsGPX2* and *OsGPX4* were up-regulated in rice stem and root tissues under drought and oxidative stress [59]. Transgenic plants overexpressing *AtGPX5* maintained good germination rate, seedling growth, and chlorophyll content under NaCl stress [22]. AtGPX5 could effectively inhibit cell death induced by two pharmacological inducers (1S, 3R)-RSL3 (RSL3) and imidazole ketone erastin (IKE), indicating that AtGPX5 could effectively protect mammalian cells from ferroptosis [60]. In our study, we found that heterologous expression of *CqGPX4* and *CqGPX15* could promote *E. coli* growth under NaCl, mannitol, CdCl_2_, and H_2_O_2_ treatment (Figure 8). All these findings provide strong evidence that the *CqGPX* genes play an important role in quinoa development and response to stress.

## 5. Conclusions

This study comprehensively identified 15 members of the glutathione peroxidase (GPX) gene family in quinoa using bioinformatics methods and analyzed their protein characteristics, chromosome distribution, gene structure, conserved domain structure, cis-acting elements, tissue-specific expression, and expression patterns under stresses. The *CqGPX* genes were specifically expressed in different tissues, and most of the *CqGPX* genes were up-regulated under salt stress, drought stress, CdCl_2,_ and H_2_O_2_ treatment, indicating that *CqGPX* genes may play an important role in quinoa in response to a variety of stresses. The activity of glutathione peroxidase enzymes in quinoa increased under stresses, and overexpression of *CqGPX4* and *CqGPX15* enhanced the stress tolerance of prokaryotic *E. coli*. This study provides an important reference for further exploring the function of *CqGPX* genes in quinoa. Obviously, additional studies based on transgenic technology, gene knocking down technology, direct ROS quantification, and detailed antioxidant assays are needed to further understand the function of *CqGPX* genes in plants.

## Figures and Tables

**Table 1:** Basic information on the quinoa *CqGPX* gene family and physicochemical properties of its encoded proteins.

Gene Name	Gene ID	Chromosome Location	Number of Amino Acid Residues	Molecular Weight (KDa)	Isoelectric Point	Instability Coefficient	Hydrophilicity Coefficient	Subcellular Localization
*CqGPX1*	AUR62024797	Chr01: 46337198–46340575	241	26.49	8.79	32.42	−0.196	Chloroplasts, mitochondria
*CqGPX2*	AUR62032425	Chr04: 52843865–52847436	235	25.85	8.81	30.2	−0.137	Chloroplasts, mitochondria
*CqGPX3*	AUR62002981	Chr06: 14361837–14364180	171	19.55	9.33	25.62	−0.269	Chloroplasts, mitochondria
*CqGPX4*	AUR62036788	Chr07: 52295212–52298236	171	19.08	9.3	26.59	−0.33	mitochondrion
*CqGPX5*	AUR62002269	Chr07: 65062016–65067011	199	22.37	6.6	42.34	−0.063	Chloroplasts, mitochondria
*CqGPX6*	AUR62015425	Chr11: 20062320–20067149	200	22.56	7.63	41.98	−0.137	Chloroplasts, mitochondria
*CqGPX7*	AUR62010667	Chr13: 9382496–9384086	141	16.05	4.54	34.93	−0.156	Chloroplasts, mitochondria
*CqGPX8*	AUR62010585	Chr13: 10828050–10834294	455	50.87	5.22	42.6	−0.225	Chloroplasts, mitochondria
*CqGPX9*	AUR62010584	Chr13: 10861209–10865017	234	26.19	8.61	37.64	−0.317	Chloroplasts, mitochondria
*CqGPX10*	AUR62035236	Chr14: 7766701–7773388	323	36.17	9.43	34.67	−0.416	nucleus
*CqGPX11*	AUR62005465	Chr14: 52044958–52047589	141	15.98	9.06	42.97	−0.415	mitochondrion
*CqGPX12*	AUR62017227	Chr16: 68266257–68269618	170	18.91	6.73	30.86	−0.221	Chloroplasts, mitochondria
*CqGPX13*	AUR62017225	Chr16: 68316179–68319386	235	26.13	8.82	38.37	−0.311	Chloroplasts, mitochondria
*CqGPX14*	AUR62033706	Chr17: 53972176–53976971	171	19.04	9.29	28.3	−0.333	mitochondrion
*CqGPX15*	AUR62033705	Chr17: 53998828–54003613	171	19.04	9.29	28.3	−0.333	mitochondrion

## Data Availability

The data presented in this study are available in the article and Supplementary Materials.

## Supplementary Materials

The following supporting information can be downloaded at: https://www.mdpi.com/article/10.3390/antiox14080940/s1, Figure S1: Amino acid sequence alignment of CqGPX proteins; Table S1: The *GPX* genes from other plant species selected for phylogenetic tree construction; Table S2: Analysis of the 10 conserved motifs of CqGPX proteins in *C. quinoa*; Table S3: Segmentally duplicated *CqGPX* gene pairs; Table S4: Information of cis-element in CqGPXs promoter region; Table S5: qRT-PCR and gene cloning primers.
