# Supplementary material for: The Glutathione Peroxidase Gene Family in Chenopodium quinoa: Genome-Wide Identification, Classification, Gene Expression and Functional Analysis
Source: Antioxidants (Basel). 2025 Jul 30;14(8):940. doi: 10.3390/antiox14080940 (PMC12382766; doi:10.3390/antiox14080940)
Supplement: Supplementary file 1 [file antioxidants-14-00940-s001.zip › Figure S1 Amino acid sequence alignment of CqGPX proteins.pdf]

```
CqGPX3     ..................................................      0
CqGPX11    ..................................................      0
CqGPX9     ..................................................      0
CqGPX8     ..................................................      0
CqGPX7     ..................................................      0
CqGPX1     ..................................................      0
CqGPX6     ..................................................      0
CqGPX10    MVAILGPDPSHPNPSSSLTVKPEPQPRFSSKAADLNVESLKNGNSEQVSS     50
CqGPX4     ..................................................      0
CqGPX13    ..................................................      0
CqGPX12    ..................................................      0
CqGPX2     ..................................................      0
CqGPX15    ..................................................      0
CqGPX14    ..................................................      0
CqGPX5     ..................................................      0


CqGPX3     ..................................................      0
CqGPX11    ..................................................      0
CqGPX9     .......MLRSSIFSLFRQRSIPISLVSIVSFNQLSSISKPTHFRSSINL     43
CqGPX8     ..................................................      0
CqGPX7     ..................................................      0
CqGPX1     .MAFSSIFTSPLPSLSISYARNRPNFSPSMALHSISSNLGFASLNSSFFQ     49
CqGPX6     ..............................................MMAFGKLT      8
CqGPX10    VVPNVGIPAHEHISPACAKGAYPNGYCGFVHVEDLAAAHILAMEESKASG    100
CqGPX4     ..................................................      0
CqGPX13    .......MLRSSIFSLFRHRSIPISLVSVVSYNQSSSISKPTHFRSSINL     43
CqGPX12    ..................................................      0
CqGPX2     .MAFSSIFTSPLPSLSISNARIRPNFSPFMALHSISSKIGFGSLNSSFFQ     49
CqGPX15    ..................................................      0
CqGPX14    ..................................................      0
CqGPX5     ..............................................MAFGILT      7


CqGPX3     ......................MATYYSYAQ.......PRSVHEFIVK.     19
CqGPX11    ..................................................      0
CqGPX9     DNLSSEYFYHSP..FSQSYSQKSMASDSSAQ........PKSVHDFVVK.     82
CqGPX8     ......................MAADSSQP.........KSVHEFVVK.     17
CqGPX7     ......................MASQPTET........PQSIYNFTIK.     18
CqGPX1     DGFSLKSLNFNG.VSVKSQNSISSGVYARAAT.......EQTIHDYTVK.     90
CqGPX6     HWVSLLFLGFAVTLFYIYPSLSSQQSPEELP.........NSIYDFTVK.     48
CqGPX10    RYICSSSVAHWSDVLAGLRAKYPMYPFETKPNNNEGDRNQHSIDASMLLR    150
CqGPX4     ......................MGAVESVP........QQSIHEFTVK.     18
CqGPX13    DHLSSGYLNHSP.FSQSFSSQKSMASDSSVQ........PKSVHDFVVK.     83
CqGPX12    ......................MAADSSQP.........RSVHEFVVK.     17
CqGPX2     DGFSLKSFNFNG.VSVKSQNSISAGVYARAAT.......EQTIHDFTVK.     90
CqGPX15    ......................MGAVESVP........QQSIHEFTVK.     18
CqGPX14    ......................MGAVESVP........QQSIHEFTVK.     18
CqGPX5     HWVSLLFLGFALTLFYIYPSLSSQQSAEELP.........NSLYDFTVK.     47


CqGPX3     ..........DASGKDVNLSIYKGKVLLIINVASLCGLTKKNYTYLTQLY     59
CqGPX11    ......................MVPFEYKPYLDSKTLNRNFAYMRSLY     26
CqGPX9     ..........DARGNDVDLSIYKGKVLLIVNVASQCGLTNSNYTELTELY    122
CqGPX8     ..........DASGNDVDLSIYKGKVLLILNVASLCGLSNTNYKELTQLY     57
CqGPX7     ..........DAKGNDVKLSDYKGKVLLIVNVASKCGMTNSNYTELNQLY     58
CqGPX1     ..........DIDGNDVALSKFKGRVLLCVNVASKCGLTSSNYSELSHLY    130
CqGPX6     ..........DINGKNVSLSTYKGKVLLIVNVASQCGLTKSNYHEMNILY     88
CqGPX10    FCSWGYKVSKDYRGKDVDLSIYKGKVLLIVNVATKCGFTNIQLPQLTELY    200
CqGPX4     ..........DNRGKDVDLSMYKGKVLLVVNVASKCGFTNVNYTQLTELY     58
CqGPX13    ..........DARGNDVDLSVYKGKVLLIVNVASQCGLTNSNYAELTELY    123
CqGPX12    ..........DASGNDVDLSIYKGKVLLILNVASLCGLSNTNYTELTQLY     57
CqGPX2     ..........DIDGNDVALSKFKGKVLLCVNVASKCGLTSSNYSELSHLY    130
CqGPX15    ..........DNKGKDVDLSMYKGKVLLVVNVASKCGFTNVNYTQLTELY     58
CqGPX14    ..........DNKGKDVDLSMYKGKVLLVVNVASKCGFTNVNYTQLTELY     58
CqGPX5     ..........DINGKNVSLSTYKGKVLLIVNVASQCGLTKSNYQELNILY     87
```

Signature 1

```
CqGPX3     DKYKDLGFEILAFPSNQFK.QEPGTNEEIVEFACTNYKAEFPIFDKIEVN    108
CqGPX11    KKYKDQ.....AFPCNQFGWQEPGSNEEINEAVYSSFKAEFPLFQKIDVN     71
CqGPX9     EKYKDQGFEILAFPCNQFGNQEPGSNEEIVQFACTRFKAEYPIFDKVEVN    172
CqGPX8     DKYKGLGLEIFAFPCNQFHNQEPGTNEEIVELACTKFKAEFPIFDKIEVN    107
CqGPX7     DKYKDQGLEILAFPCNQFGDEEPGSNDEIIEFVCTRFKSDFPIFDKIEVN    108
CqGPX1     EKYKTQGFEILAFPCNQFGGQEPGSNPEIKNFACTRFKAEFPIFDKVDVN    180
CqGPX6     KKYKDQGFEILAFPCNQFGWQEPGSNEEINEAVCSSFKAEFPLFQKIDVN    138
CqGPX10    RKYKDQGFQVLAFPCNQFLKQSPGSSEKTRKVACDRFKAEYPVFRKVCVN    250
CqGPX4     QKYRDNGFEVLAFPCNQFLKQEPGTSEDAANFACTRFSAEYPIFQKVCVN    108
CqGPX13    EKYKDQGFEILAFPCNQFGNQEPGSNEEIVQFACTRFKAEYPIFDKVEVN    173
CqGPX12    DKYKGLGLEILAFPCNQFHNQEPGTNEEIVELACTKFKAEFPIFDKIEVN    107
CqGPX2     EKYKTQGFEILAFPCNQFGGQEPGSNPEIKNFACTRFKAEFPIFDKVDVN    180
CqGPX15    QKYRDNGFEVLAFPCNQFLKQEPGTSEDAANFACTRFSAEYPIFQKVCVN    108
CqGPX14    QKYRDNGFEVLAFPCNQFLKQEPGTSEDAANFACTRFSAEYPIFQKVCVN    108
CqGPX5     KKYKDQGFEILAFPCNQFGWQEPGSNEEINEAVCSSFKAEFPLFQKIDVN    137
```

Signature 2

```
CqGPX3     GSNAAPIFKYLKSTKRG...FMGDRIKWNFTKFVVDKHGNVVDRISPFTP    155
CqGPX11    GKNAAPLYKFLKSKKTG...VIVDETKWNFAKILVDRQGSVVERYAPVTS    118
CqGPX9     GGNTAPIYKFLKSSKGG...FLGSDIKWNFSKFLVDKDGNVVDRYAPTTS    219
CqGPX8     GSNAAPIFKYLKSSKSG...FLGDGIKWNFTKFLVDRDGVVVDRYSPFTS    154
CqGPX7     GDNASPLYQFLKLGKWG...IFGDDIQWNFAKFLVDKDGQVVGRYYPTTS    155
CqGPX1     GPNTAPVYQFLKSSAGG...FFGDLIKWNFEKFLVDKNGKVVERYPPTTS    227
CqGPX6     GKNAAPLYKYLKSKKSG...VIVDEIKWNFAKFLVDRQGSVVERYAPVTS    185
CqGPX10    GPKTPPLYKFLKASKINGETKWANRVKWNFTKFLIDVDGQVIYRRGLTTA    300
CqGPX4     GPKTAPVYKFIKASKRS..SLFGNSVKWNFTKFLVGKDGKVIKRYGTTTP    156
CqGPX13    GSNTAPIYKFLKSSKGG...FLGSDIKWNFSKFLVDKDGNVVDRYAPTTS    220
CqGPX12    GSNAAPIFKYLKSNKSG...FLGDGIKWNFTKFLVDRDGVVVDRYSPFTS    154
CqGPX2     GPNTAPVYQFLKSSAGG...FFGDLVKWNFEKFLVDKNGKVVERYPPTTS    227
CqGPX15    GPKTAPVYKFLKASKRS..SLFGNSVKWNFTKFLVGKDGKVVKRYGTTTP    156
CqGPX14    GPKTAPVYKFLKASKRS..SLFGNSVKWNFTKFLVGKDGKVVKRYGTTTP    156
CqGPX5     GKNAAPLYKFLKSKKSG...VIVDEIKWNFAKFLVDRQGSVVERYAPVTS    184
```

Signature 3

```
CqGPX3     LKIVEK......DLKKLLGVPK.....................................    171
CqGPX11    PLQIEAKPNAG.SVKRASALQLQ.....................................    140
CqGPX9     PKSIEK......DVKKLLGIA.......................................    234
CqGPX8     PKSIEPTNSPRKTIKSPFFYRLQPNPKLASHNHHTSLITFHSLTLPPVSG         204
CqGPX7     PFSLEY......DIKRLLEIS.......................................    170
CqGPX1     PIQIEK......DIQKLLAA........................................    241
CqGPX6     PLQIES......EVQNLLGSS.......................................    200
CqGPX10    PFKMER......QIVKALEERAARQQIMY...............................    323
CqGPX4     PLSIEG......DIKKALGEM.......................................    171
CqGPX13    PKSIEK......DVKKLLGIA.......................................    235
CqGPX12    PKSIEK......DLKKLLGIPK......................................    170
CqGPX2     PIQIEK......DIQKLLAA........................................    241
CqGPX15    PLSIEG......DIKKALGEM.......................................    171
CqGPX14    PLSIEG......DIKKALGEM.......................................    171
CqGPX5     PLQIES......EIQNLLGSS.......................................    199


CqGPX3     .....................................................    171
CqGPX11    .....................................................    140
CqGPX9     .....................................................    234
CqGPX8     LPAGAETTNDVSPSTWPQLMDAMDMIRDTIDAHLSYLKPDFVFYDLAFWL    254
CqGPX7     .....................................................    170
CqGPX1     .....................................................    241
CqGPX6     .....................................................    200
CqGPX10    .....................................................    323
CqGPX4     .....................................................    171
CqGPX13    .....................................................    235
CqGPX12    .....................................................    170
CqGPX2     .....................................................    241
CqGPX15    .....................................................    171
CqGPX14    .....................................................    171
CqGPX5     .....................................................    199


CqGPX3     .....................................................    171
CqGPX11    .....................................................    140
CqGPX9     .....................................................    234
CqGPX8     PEVASKHSLQTTSLRRLTEPPPNFPSSAVRLRLFEAQAQSYLYKQEFGGG    304
CqGPX7     .....................................................    170
CqGPX1     .....................................................    241
CqGPX6     .....................................................    200
CqGPX10    .....................................................    323
CqGPX4     .....................................................    171
CqGPX13    .....................................................    235
CqGPX12    .....................................................    170
CqGPX2     .....................................................    241
CqGPX15    .....................................................    171
CqGPX14    .....................................................    171
CqGPX5     .....................................................    199


CqGPX3     .....................................................    171
CqGPX11    .....................................................    140
CqGPX9     .....................................................    234
CqGPX8     ISFTERITISCDAADALAAKACKEMEGTYCQFYQQILQKPVLLIGPVVPE    354
CqGPX7     .....................................................    170
CqGPX1     .....................................................    241
CqGPX6     .....................................................    200
CqGPX10    .....................................................    323
CqGPX4     .....................................................    171
CqGPX13    .....................................................    235
CqGPX12    .....................................................    170
CqGPX2     .....................................................    241
CqGPX15    .....................................................    171
CqGPX14    .....................................................    171
CqGPX5     .....................................................    199


CqGPX3     .....................................................    171
CqGPX11    .....................................................    140
CqGPX9     .....................................................    234
CqGPX8     PPASKLDDRFDCWLKEFGRASVIYCALGSDCILEKDQFQELFLGMELTVD    404
CqGPX7     .....................................................    170
CqGPX1     .....................................................    241
CqGPX6     .....................................................    200
CqGPX10    .....................................................    323
CqGPX4     .....................................................    171
CqGPX13    .....................................................    235
CqGPX12    .....................................................    170
CqGPX2     .....................................................    241
CqGPX15    .....................................................    171
CqGPX14    .....................................................    171
CqGPX5     .....................................................    199


CqGPX3     .....................................................    171
CqGPX11    .....................................................    140
CqGPX9     .....................................................    234
CqGPX8     QFINARLMSIELKVGVEVERQDDDGYFTKSDVANPVSTVMEAESVDGKEV    454
CqGPX7     .....................................................    170
CqGPX1     .....................................................    241
CqGPX6     .....................................................    200
CqGPX10    .....................................................    323
CqGPX4     .....................................................    171
CqGPX13    .....................................................    235
CqGPX12    .....................................................    170
CqGPX2     .....................................................    241
CqGPX15    .....................................................    171
CqGPX14    .....................................................    171
CqGPX5     .....................................................    199
```
