# Supplementary material for: The Glutathione Peroxidase Gene Family in Chenopodium quinoa: Genome-Wide Identification, Classification, Gene Expression and Functional Analysis
Source: Antioxidants (Basel). 2025 Jul 30;14(8):940. doi: 10.3390/antiox14080940 (PMC12382766; doi:10.3390/antiox14080940)
Supplement: Supplementary file 1 [file antioxidants-14-00940-s001.zip › Captions for SUPPLEMENTARY MATERIAL.docx]

**Figure S1.** Amino acid sequence alignment of CqGPX proteins. The rectangles indicate the three highly conserved regions (signature1-3), Conserved potential catalytic residues labeled with solid circles, catalytic residues Cys (C), Gln (Q), Trp (W), and Asn (N).

**Table S1** The *CqGPX* genes from other plant species selected for phylogenetic tree construction.

**Table S2** Analysis of the 10 conserved motifs of CqGPX proteins in C.quinoa.

**Table S3** Segmentally duplicated *CqGPX* gene pairs.

**Table S4** Information of cis-element in *CqGPXs* promoter region.

**Table S5** qRT-PCR and gene cloning primer.
